# HARCI-EU, a harmonized gridded dataset of critical infrastructures in Europe for large-scale risk assessments

**DOI:** 10.1038/s41597-019-0135-1

**Published:** 2019-07-18

**Authors:** Filipe Batista e Silva, Giovanni Forzieri, Mario Alberto Marin Herrera, Alessandra Bianchi, Carlo Lavalle, Luc Feyen

**Affiliations:** European Commission, Joint Research Centre, Ispra, Italy

**Keywords:** Natural hazards, Climate-change adaptation, Climate-change impacts, Geography

## Abstract

Critical infrastructures (CIs) are assets, systems, or parts thereof that are essential for the maintenance of socioeconomic functions, health, safety and well-being of people. The exposure of CIs to natural and man-made hazards poses a risk to the economy and society. The spatial distribution of CIs and their economic value are a prerequisite for quantifying risk and planning suitable protection and adaptation measures. However, the incompleteness and inconsistency of existing information on CIs hamper their integration into large-scale risk frameworks. We present here the ‘HARmonized grids of Critical Infrastructures in EUrope’ (HARCI-EU) dataset. It represents major CIs in the transport, energy, industry and social sectors at 1 km^2^ expressed in sector-specific, economically-relevant units. The HARCI-EU grids were produced by integrating geospatial and statistical data from multiple sources. Correlation analysis performed against independent metrics corroborates the approach showing average Pearson coefficients ranging between 0.61 and 0.95 across the sectors. HARCI-EU provides a consistent mapping of CIs in key sectors that can serve as exposure information for large-scale risk assessments in Europe.

## Abstract (summary)

**Table:** 

Design Type(s)	data integration objective • modeling and simulation objective
Measurement Type(s)	Infrastructure
Technology Type(s)	digital curation
Factor Type(s)	Sector • geographic location
Sample Characteristic(s)	Europe • anthropogenic habitat

Machine-accessible metadata file describing the reported data (ISA-Tab format)

## Background & Summary

Critical infrastructures (CIs) are physical or virtual assets or systems of assets that are vital to ensuring health, well-being and security of people and whose disruption or destruction may undermine communities or countries at large^1,2^. They include (and are not limited to) infrastructure related to transport, energy generation and transmission, water, industry, education and health, information and communication technology. Exposure of CIs to hazards poses a risk to economies and societies^3–5^. Recent events, such as the Eyjafjöll volcanic eruption in Iceland in 2010^6^, the Great East Japan Earthquake in 2011^7^, and Hurricane Harvey in the Unites States in 2017^8^, have shown how disruption of key systems and essential services can lead to substantial socio-economic impacts. The main threats presented by hazards to CIs include damage or destruction from extreme events^9–13^, whose effects can be exacerbated when multiple hazards co-occur^14–17^. Dependency networks of CIs may further amplify economic damages and trigger cascading failures^18^ with possible global scale effects^16,19,20^. This is of particular concern for Europe, as the severity and frequency of weather-related hazards is expected to intensify in view of climate change^21^.

The development of reliable and resilient infrastructure is among the United Nations’ Sustainable Development Goals^22^. Besides, there is increasing interest in identifying and assessing disaster risk at large scale, expressed by the Sendai Framework for Disaster Risk Reduction 2015–2030^23^ and the Decision on a European Union Civil Protection Mechanism^24^ that calls participating states to perform National Risk Assessments with periodic reporting. The aim of the latter is to promote an effective and coherent approach to prevention of and preparedness for disasters.

Risk assessment requires the integration of hazard, exposure and vulnerability^25^. The hazard represents the agent that may affect CIs, exposure refers to the spatial distribution of CIs and their associated services exposed to the hazard, and vulnerability expresses the propensity of CIs to be affected by the hazard. Typically, a risk assessment consists in overlaying geospatial information on infrastructures and key socioeconomic assets with hazard maps.

Information on the spatial distribution of CIs is, therefore, a prerequisite for quantifying hazard risk to CIs and planning suitable risk reduction measures in order to safeguard CIs and ultimately secure the functioning of societies^26^. However, geospatial data on CIs is often incomplete and scattered across multiple and inconsistent data sources, thus hampering their integration in large-scale risk frameworks. The European Pollutant Release and Transfer Register (E-PRTR), for instance, contains the location of industrial, energy and waste treatment facilities. Its original scope was to monitor emissions of pollutants from the main emitters; hence, facilities whose emissions levels fall under a certain threshold are not included, regardless of their economic importance. Other sources, such as the voluntary geographical information project Open Street Map (OSM), or the proprietary navigation dataset TomTom Multinet miss many features of the real world, especially those deemed less interesting to the average user. Data completeness differs between data sources and across domains or geographical areas within the same data source. Furthermore, information on data quality and completeness often does not exist due to the lack of benchmarks and validation efforts.

Data inconsistency may arise in various ways: different nomenclatures and/or mapping criteria across data sources or types of critical infrastructures. For example, transport infrastructure can be represented in a Geographical Information System (GIS) using alternative data structures: roads or railways are typically represented by line segments, while ports and airports by points or polygons. Such variety in format and spatial representation raises a series of technical problems for their use in a common risk assessment framework. How, for example, can a port represented as point feature in a GIS be compared to 1 km of road? How can 1 km of motorway be compared to 1 km of local road? How can a metal industry be compared to a refinery, or a hospital to a school? In order to compare impacts of a given hazardous event on different infrastructure types and sectors using a consistent methodology applicable at large scale, there is need for harmonized exposure information.

Here, we describe and make publicly available the ‘HARmonized grids of Critical Infrastructures in EUrope’ (HARCI-EU)^27^, employed in a previous study to quantify future risks to CIs in Europe due to climate extreme events^5^. To solve the referred data completeness and inconsistency issues, we integrate CIs-relevant geospatial data from state-of-the-art sources with national-scale statistics of their productivity or use. HARCI-EU is a novel, coherent representation of CIs in Europe, consisting of 22 grid maps at 1 km spatial resolution, covering the transport, energy, industry and social sectors. Each map represents the spatial distribution of a given infrastructure type expressed in sector-specific economic units.

According to the relevant European Directive^1^, CIs in the energy and transport sectors were deemed priority for their identification, designation and protection. Although HARCI-EU goes beyond these two sectors alone, it is not a complete account of all possible CIs in existence. While there is no ultimate list or classification of CIs, the United States Presidential Policy Directive on CI Security and Resilience, for example, cites 16 sectors of CIs, some of which are not included in HARCI-EU (e.g. defence, food, finance, water supply)^28^. Notwithstanding, the high spatial and thematic resolution and coverage make HARCI-EU a useful exposure dataset for assessing the risks of hazards to critical infrastructures in Europe. Future developments should focus on expanding HARCI-EU to encompass further CI categories.

## Methods

**Fig. 1:**
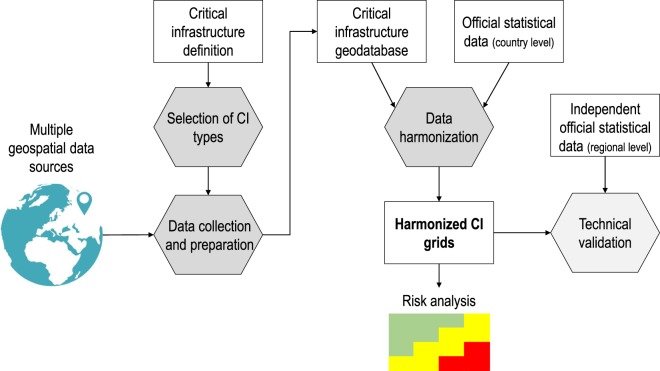
General workflow of the production and validation of the HARCI-EU dataset.

We structured the production of the harmonized grids of CIs in three main phases as shown in the workflow chart in Fig. 1: (a) selection of CI types; (b) data collection and preparation; and (c) data harmonization.

We used the definition of CI mentioned earlier to guide the selection of infrastructure types. Given the broad definition of CI, we constrained the selection to physical infrastructures for which data were more readily available and which are more likely to be exposed to physical threats such as natural hazards, while fulfilling the priorities set by the relevant European Directive (i.e. energy and transport sectors)^1^. Therefore, we considered various infrastructures in four key sectors as follows: transport, energy, industry, and social infrastructure. We further subdivided each sector in two or more subsectors, with each subsector containing one or more specific infrastructure types (see Table 2 in the Data Records section for the full classification).

A significant part of this study was devoted to collecting detailed geospatial information of current CIs from multiple data sources. Information on CIs in Europe was rather scattered and mixed, with alternative sources for different infrastructure types, as well as for the same infrastructure type. We browsed for potential datasets, and selected the most suitable candidate for each infrastructure category based on following criteria:

- Geographical coverage: European datasets were preferred over national or worldwide ones, in order to avoid inconsistent data across countries or too low detail, respectively.
- Data completeness: the highest data completeness was preferred.
- Data consistency: datasets with transparent and consistent mapping and reporting methodologies.
- Spatial resolution: highest possible.
- Data update: most recent.
- Thematic coverage: datasets covering the largest possible number of infrastructure types within a sector.

In total, we resorted to seven data sources, with reporting years between 2010 and 2014. A geo-database was constructed from them according to the structure and characteristics laid down in Table 2 in the Data Records section. The spatial information is stored in vector format as either points or polylines, depending on the infrastructure type, and covering the EU28 + EFTA countries (i.e. Iceland, Lichtenstein, Norway and Switzerland).

In the data harmonization phase, infrastructure types belonging to the same sector were expressed in a common measurement unit, so that their relative importance can be evaluated. In that process, we adopted the following principles:

- The total value of assets being considered within a country or region is not affected by missing CI locations;
- Each CI is quantitatively evaluated at its geographical location and expressed in a sector-specific economic unit, as a proxy of its societal value/usefulness.

The data harmonization consisted of a two-fold data transformation. First, we converted vector data (points and polylines) to gridded data (i.e. raster) with a cell size of 1 km x 1 km. This conversion is instrumental for impacts models, as they typically work with gridded data structures. The spatial resolution of the output was chosen to properly represent the spatial distribution of CIs at a level of detail suitable for country or continent-wide risk assessments.

Subsequently, we assigned to each raster cell an ‘intensity’ score reflecting its relative economic value. Given the large scale scope, there is no sufficient information to characterize each CI in terms of its actual capital stock value expressed in a common currency. Therefore, our approach was to proxy the economic value of each CI using a sector-specific metric of its use: annual freight transported (transport), energy produced or transported (energy), annual turnover (industry) and annual expenditure (social) (see Table 1). This approach has already been successfully applied to estimate the economic damages to CIs due to climate hazards^5^. The referred economic variables have been collected from Eurostat at national level for the five most recent years available (typically 2009–2013). Values were averaged over the 5 year time window in order to minimize eventual outliers in the time series and the resulting values were finally assigned to infrastructures.

**Table 1:** Economic variables and units used per sector.

Sector	Economic variable	Unit
Transport infrastructure	Annual freight transported	k tonnes
Energy infrastructure	Annual energy produced/transported	k tonnes oil equivalent
Industry infrastructure	Annual turnover	Million EUR
Social infrastructure	Annual expenditure	Million EUR

Depending on the type of infrastructure, we used two methods to assign economic values to infrastructure locations:

- Direct assignment
- Downscaling

The direct assignment is the most accurate method, but was applicable only for ports and airports, for which Eurostat reports annual freight transported specifically for each site. The downscaling procedure, on the other hand, consists of disaggregating the total national economic value associated with the operation of each infrastructure typology to the locations (i.e. cells) of the relevant infrastructures within the respective country. For example, the total electricity production from gas power plants of a country was disaggregated over all 1 km^2^ cells containing gas power plants located in the given country. In this process, the share of the national values assigned to each cell is proportional to the relative size or use of the local infrastructure in the country total.

The generic downscaling procedure is described by equation 1:

1 $${I}_{j,i}={V}_{j,c}\ast \left(\frac{{w}_{j,i}}{{\sum }_{i}\,{w}_{j,i}}\right)$$

where:

*I*_*j,i*_ = economic value of infrastructure type *j* in a 1 km^2^ pixel *i*

*V*_*j,c*_ = economic value related to the operation of infrastructure *j* in country *c*, as reported by Eurostat

*w* = weight = *f(j)*

for *j* = roads, *w*_*i,j*_ = length_*i,j*_ * capacity_*i,j*_ * population_*i,j*_, where length is the sum of the length of all road segments within *i*, capacity is a score of potential load of vehicles, and depends on the type of road (motorways = 5, national roads = 3, local roads = 2), and population is a proxy for the number of users, calculated as the number of residents within a 20 km radius around *i*.

for *j* = rails or inland water ways, *w*_*i,j*_ = length_*i,j*_ * average transport flow_*i,j*_

for *j* = energy production, *w*_*i,j*_ = installed capacity_*i,j*_

for *j* = electricity grid, *w*_*i,j*_ = length_*i,j*_ * voltage_*i,j*_

for *j* = gas pipelines, *w*_*i,j*_ = length_*i,j*_ * pipeline diameter_*i,j*_

for *j* = industry, *w*_*i,j*_ = number of facilities_*i,j*_

for *j* = social infrastructure, *w*_*i,j*_ = potential users_*i,j*_ = population_*i*_ / number of facilities_*i*_, where population is a proxy for the number of users, and number of facilities is the number of schools or hospitals. Both terms are calculated within a radius of 20 km around *i*, as a potential service area.

The number of residents at pixel level was taken from a high-resolution European population density map^29^. The average transport flows for railways and inland waterways were estimated by the model Transtools II^30^. The weight parameters for energy infrastructures (i.e. installed capacity, voltage and pipeline diameter) were available from the Platts database (https://www.spglobal.com/platts). We recognize that such approach introduces potential sources of subjectivity, such as the choice of type and number of predictors and their combination in the weighting functions. The technical validation, described in the dedicated section later on, addresses this issue and supports the identified functions.

A key advantage of the proposed harmonization approach is that it converts the original categorical information in comparable economic terms, and allows the summation of economic value of different infrastructure types *j* of the same sector *s*, as expressed in equation 2.

2 $${I}_{s,i}=\sum _{j}\,{I}_{i,j}\,with\,j\,\varepsilon \,s$$

## Data Records

The HARCI-EU dataset corresponds to the final output of the data harmonization procedure as described in the Methods section and is publicly available from the Figshare repository^27^. The HARCI-EU dataset contains 22 grids in GeoTIFF format with a resolution of 1 km^2^. The grids use the ETRS89 coordinate system and the Lambert Azimuthal Equal Area map projection. They represent values in sector-specific economic units (see Table 1) ranging between zero (i.e. absence of infrastructure) and CI-specific maximum value resulting from the downscaling procedure described in the Methods section. Raster cells outside the area of interest have null values. These files are best visualized and manipulated using appropriate GIS software.

**Fig. 2:**
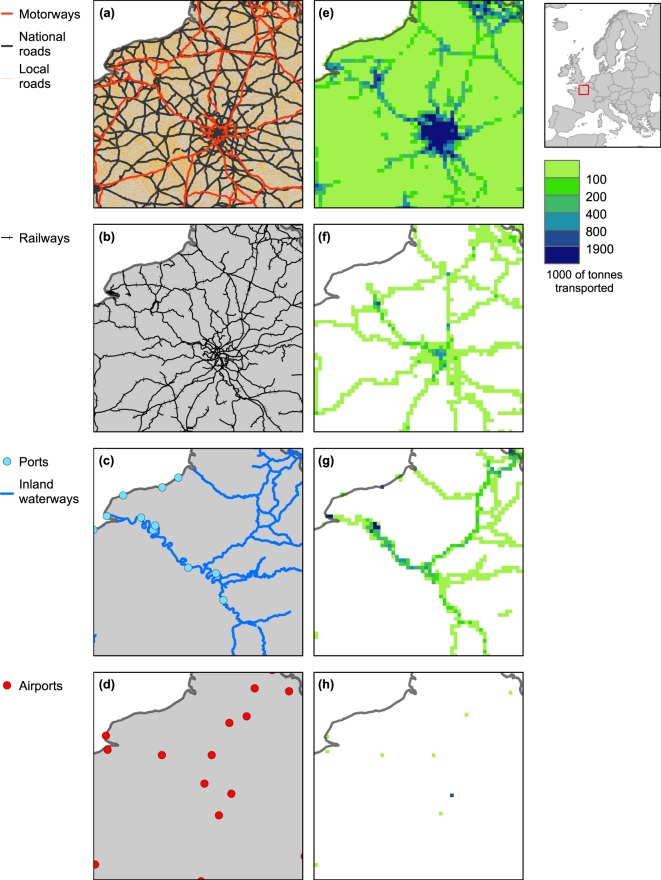
Location and economic value of various transport infrastructure types around Paris, France, according to HARCI-EU. Panels (a) to (d) show infrastructures represented in the original vector format and panels (e) to (h) show the corresponding harmonized grids at 1 km resolution.

**Fig. 3:**
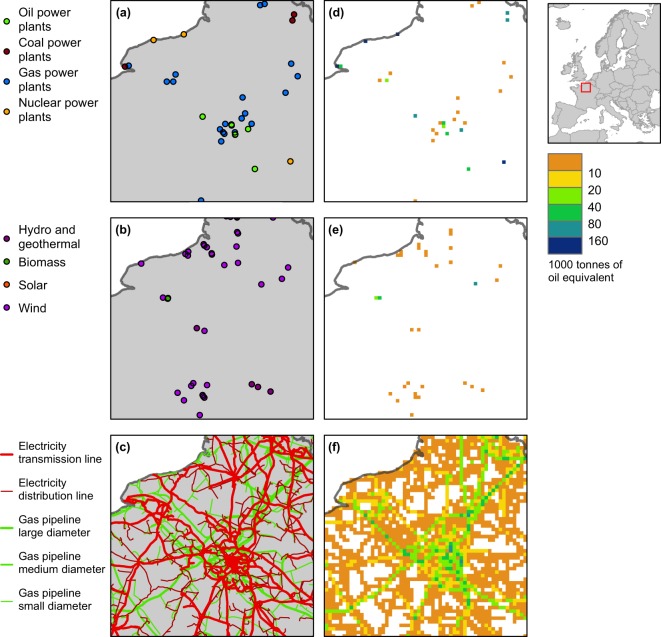
Location and economic value of various energy infrastructure types around Paris, France, according to HARCI-EU. Panels (a) to (c) show infrastructures represented in the original vector format and panels (d) to (f) show the corresponding harmonized grids at 1 km resolution.

**Fig. 4:**
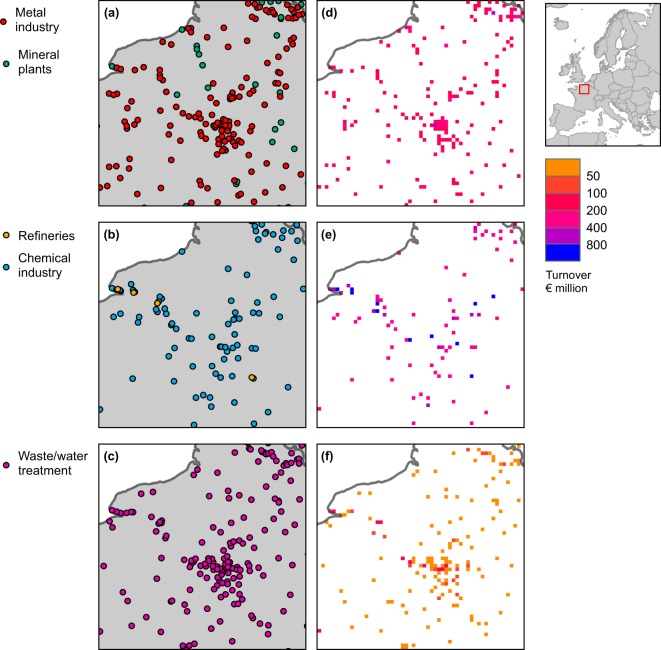
Location and economic value of various industry infrastructure types around Paris, France, according to HARCI-EU. Panels (a) to (c) show infrastructures represented in the original vector format and panels (d) to (f) show the corresponding harmonized grids at 1 km resolution.

**Fig. 5:**
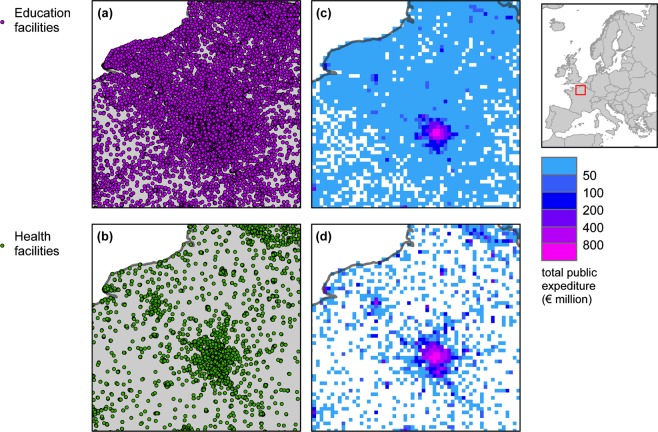
Location and economic value of various social infrastructure types around Paris, France, according to HARCI-EU. Panels (a,b) show infrastructures represented in the original vector format and panels (c,d) show the corresponding harmonized grids at 1 km resolution.

Table 2 indicates for each infrastructure type the sector and sub-sector it belongs to, the original data structure type, sources used, reference data and raster filename. The items referring to ‘local roads’, ‘roads of national importance’ and ‘motorways’ are merged in one single raster file representing the whole road network. Figures 2–5 show extracts of the original vector data and the corresponding harmonized grids for the geographical area around Paris, France.

**Table 2:** List of infrastructures used in this study, sources used, reference dates, and raster filenames.

Sector	Sub-sector	Infrastructure type	Data structure	Source	Source description	Reference date	Raster filename^27^
Transport	Roads	Local roads	Vector (lines)	Open Street Map (http://download.geofabrik.de)	Voluntary Geographic Information	2014	ci_tra_01.tif
		Roads of national importance					
		Motorways					
	Other transport networks	Railways	Vector (lines)				ci_tra_02.tif
		Inland waterways		UNECE (https://www.unece.org/trans/main/sc3/maps.html) + EuroRegionalMap (https://eurogeographics.org/products-and-services/euroregionalmap)	Public (UNECE); Proprietary (EuroRegionalMap)	2013	ci_tra_03.tif
		Ports	Vector (points)	CORINE Land Cover (CLC) (https://land.copernicus.eu/pan-european/corine-land-cover) + EuroRegionalMap (https://eurogeographics.org/products-and-services/euroregionalmap)	Public (CLC); Proprietary (EuroRegionalMap)	2012	ci_tra_04.tif
		Airports					ci_tra_05.tif
Energy	Non-renewable energy production	Coal power plants	Vector (points)	Platts (https://www.spglobal.com/platts)	Proprietary, specialized geodatabase	2013	ci_ene_01.tif
		Gas power plants					ci_ene_02.tif
		Oil power plants					ci_ene_03.tif
		Nuclear power plants					ci_ene_04.tif
	Renewable energy production	Biomass and geothermal power plants	Vector (points)				ci_ene_05.tif
		Hydro power plants					ci_ene_06.tif
		Solar power plants					ci_ene_07.tif
		Wind power plants					ci_ene_08.tif
	Energy transport	Electricity distribution / transmission	Vector (lines)				ci_ene_09.tif
		Gas pipelines					ci_ene_10.tif
Industry	Heavy industries	Metal industry	Vector (points)	E-PRTR (https://prtr.eea.europa.eu)	Public	2013	ci_ind_01.tif
		Mineral industry					ci_ind_02.tif
		Chemical industry					ci_ind_03.tif
		Refineries		Global Energy Observatory (http://globalenergyobservatory.org)	Public	2010	ci_ind_04.tif
	Water/waste treatment	Water and waste treatment	Vector (points)	E-PRTR (https://prtr.eea.europa.eu)	Public	2013	ci_ind_05.tif
Social	Education	Education facilities	Vector (points)	Open Street Map(http://download.geofabrik.de)	Open, Voluntary Geographic Information	2014	ci_soc_01.tif
	Health	Health facilities					ci_soc_02.tif

## Technical Validation

The production of the HARCI-EU grids^27^ relied on three types of data inputs:

- Location of CIs obtained from seven different sources of geospatial data;
- Economic value associated with the operation of each infrastructure type from Eurostat at country level;
- Weighting parameters for the spatial disaggregation of country volumes, based on infrastructure characteristics available from the geospatial data or auxiliary sources (e.g. gridded population, transport model).

For obtaining the location of CIs and the attributes used in the weighting functions, we carefully selected data sources and evaluated their appropriateness. A systematic, quantitative validation of each selected source is out of the scope of this study. The economic value associated with the operation of each infrastructure type was obtained from an official source of statistical data, but at a coarse spatial resolution. An additional source of uncertainty concerns the assumption of linearity between the economic value of infrastructures and the chosen weighting parameters. This key assumption governed the downscaling of country volumes of activity for each infrastructure type to grid cell level, as specified in equation 1.

The multiple sources of uncertainty and their potential propagation require a validation of the final output. Yet, the lack of alternative datasets representing the same economic metrics of HARCI-EU impedes a standard validation exercise. The approach to assess the validity of HARCI-EU therefore consisted of evaluating the plausibility of the resulting spatial distribution of CI harmonized values per sector and per sub-national units across Europe.

We used an independent source of data (Cambridge Econometrics European Regional Database, CE-ERD^31^) consisting of a time series for three key variables available at sub-national level: total population, gross value added (GVA) in the industry sector and GVA in all goods-related sectors (defined as the total GVA minus the GVA for financial and business services and non-market services). The time series spanned from 2009 to 2013 (to match temporally with the CI economic values), and the average over this period was taken. The spatial resolution corresponded to the NUTS3 level. The NUTS classification is hierarchical system of territorial units used for statistical data reporting in Europe. The NUTS3 level corresponds to country provinces or districts, and comprises 1359 regions within the area of interest (EU + EFTA), with a median size of 1724 km^2^ (NUTS3 version 2010).

Due to the importance of CIs for the socioeconomic system, the chosen independent variables at the regional level are a good benchmark to evaluate the appropriateness of the framework applied to map CIs and their economic value. To this aim, for each sector we correlated the total economic value of CIs represented in the HARCI-EU grids with the selected independent variables at NUTS3 level.

Because the total number of NUTS3 is relatively high, we were able to stratify the calculation of the Pearson correlation per country, allowing a much better insight than a single measure of fit over the whole spatial domain for each sector. Countries with few NUTS3 were grouped as follows:

- Cyprus and Greece
- Czech Republic and Slovakia
- Estonia, Latvia and Lithuania
- Italy and Malta
- France and Luxembourg
- Iceland and Norway

In all other cases, correlations were based on NUTS3 for single countries. For the energy sector, however, statistical data at regional level were limited. We were able to find regional (NUTS2 or NUTS3) values of electricity production for a sample of representative countries (France, Italy, Poland, Portugal and Slovakia). Table 3 summarizes the main characteristics of the reference data used for the validation for each sector of critical infrastructures, and Table 4 reports the results obtained for each sector.

**Table 3:** Characteristics of the reference data used for the validation for each sector of critical infrastructures.

Sector	Independent variable used for the validation	Source	Coverage	Spatial unit
Transport infrastructure	GVA in goods-related sectors	CE-ERD	EU28	NUTS3
Energy infrastructure	Electricity production	National Statistical Offices	France, Italy, Poland	NUTS2
			Portugal, Slovakia	NUTS3
Industry infrastructure	GVA in industry sector	CE-ERD	EU28	NUTS3
Social infrastructure	Population (no. of inhabitants)	CE-ERD	EU28, EFTA	NUTS3

**Table 4:** Results of the technical validation. Assessment for countries marked with’ was performed at NUTS2 level (applicable to the Energy sector only). Values marked with * have p-values < 0.01.

Country/group of countries	No. of NUTS regions	Transport	Industry	Social	Energy
		Pearson corr.	Pearson corr.	Pearson corr.	Pearson corr.
AT	35	0.911*	0.590*	0.996*	—
BE	44	0.739*	0.818*	0.986*	—
BG	28	0.945*	0.314	0.981*	—
GR_CY	52	0.987*	0.796*	0.995*	—
CZ_SK	22	0.928*	0.742*	0.970*	—
DE	412	0.853*	0.514*	0.963*	—
DK	11	0.814*	0.523	0.977*	—
EE_LV_LT	21	0.754*	0.468	0.978*	—
ES	59	0.956*	0.852*	0.989*	—
FI	19	0.991*	0.777*	0.987*	—
FR’	22	—	—	—	0.995*
FR_LU	97	0.844*	0.665*	0.991*	—
HR	21	0.732*	0.037	0.972*	—
HU	20	0.896*	0.677*	0.907*	—
IE	8	0.912*	0.798	0.977*	—
IT’	19	—	—	—	0.942*
IT_MT	112	0.880*	0.798*	0.995*	—
NL	40	0.712*	0.471*	0.989*	—
PL’	16	—	—	—	0.937*
PL	66	0.671*	0.459*	0.913*	—
PT	30	0.867*	0.612*	0.971*	0.867*
RO	42	0.786*	0.551*	0.921*	—
SE	21	0.986*	0.572*	0.997*	—
SI	12	0.785*	0.903*	0.963*	—
SK	8	—	—	—	0.986*
UK	139	0.675*	0.550*	0.917*	—
CH	26	—	—	0.986*	—
NO_IS	21	—	—	0.965*	—
**Average**		**0.847**	**0.613**	**0.970**	**0.946**

High correlation values between CI maps and the independent sources indicate that HARCI-EU grids represent properly the economic value of sector-specific assets and their regional distribution. For example, it is plausible to assume that more densely populated regions require more social infrastructure and expenditure. Similarly, higher GVA for goods-related sectors implies denser transport infrastructure and more freight transported. From the four sectors, the highest correlations were obtained for the social and energy infrastructures, with average country correlation of 0.97 and 0.95, respectively, and with individual country correlations always above 0.9 (except a slightly lower score in Portugal for the energy infrastructure). The transport sector also showed an overall very high correlation of nearly 0.85. However, this sector showed more variability in the country scores, ranging from around 0.67 in Poland and the UK to 0.95 or more in Bulgaria, Finland, Greece/Cyprus, Spain and Sweden.

Less satisfactory is the result obtained for the industry sector, with an average country correlation of 0.61, but with values as low as 0.31 in Bulgaria and 0.04 in Croatia. In Belgium, Greece/Cyprus, Spain, Ireland, Italy/Malta and Slovenia correlations are equal to or above 0.8. This outcome relates to at least two reasons: a) The source used to obtain the location of industrial and waste treatment facilities (i.e. EPRTR) focuses only on polluting facilities above a certain dimension, hence being incomplete by design. b) The fact that we considered only heavy industries and waste treatment plants as part of the industrial infrastructures, while the industrial GVA includes more industry types. In fact, the latter point may imply that, in countries with low correlation, heavy industry and waste treatment are less relevant for their total industrial output.

## Usage Notes

The data harmonization approach transformed discrete and categorical vector records of CIs into a data format with uniform representation (regular grid cells of 1 km^2^) and described by the economic value of assets with common units within each sector, enabling comparability of exposure across CI types within the same sector. It further minimized the effects of missing infrastructures in the input data sources as the total economic value of a given infrastructure type in a given country was preserved within that country. This means that even if a particular CI location is missing, the total value associated with that infrastructure type is retained within the country, enabling aggregated impacts between countries to be compared. However, it must be noted that the harmonization procedure does not prevent underestimation of exposure at site-specific level whenever infrastructure data were missing.

**Fig. 6:**
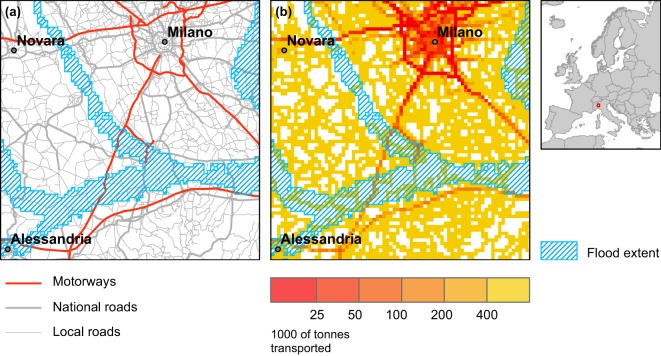
Original road vector data and HARCI-EU road layer overlaid onto a 100-year return period flood extent.

The HARCI-EU layers can be combined with hazard maps to derive an impact measured in the same unit as of the harmonized layers. The assumption is that – under a similar vulnerability scenario (CI- and hazard-specific) – locations with higher economic value are associated with higher impacts in case of a hazardous event. Translating impact into potential monetary losses is possible by applying cost coefficients (or cost curves) that link estimated impacts (harmonized layers * hazard) with actual observed losses due to hazards, as applied by Forzieri *et al*.^5^ for climate related hazards. Figure 6 shows an example of original road data and its respective harmonized version overlaid onto a flood extent^32^.

More sophisticated risk assessment approaches may account for ‘network effects’, such as when the disruption of a network segment or node affects a wider service area (spatial spillovers), as well as ‘cascading effects’, i.e. chain of negative events triggered by an initial disruption in a system, possibly resulting in sector spillovers. While the HARCI-EU layers are readily applicable for straightforward, overlay-based risk assessments, their integration in complex system modelling to account for the referred effects may require further elaboration.

The quality of the exposure layers affects the reliability of the final risk estimates. While it is not feasible to systematically assess the accuracy of every input and for every CI type, the technical validation carried out supports the overall plausibility of the resulting HARCI-EU layers for risk assessment at least at mesoscale (e.g. sub-national) and particularly for transport, energy and social CIs.

## ISA-Tab metadata file

Download metadata file
